# A repeated cross-sectional study of socio-economic inequities in dietary sodium consumption among Canadian adults: implications for national sodium reduction strategies

**DOI:** 10.1186/1475-9276-13-44

**Published:** 2014-06-05

**Authors:** Lindsay McLaren, Shayla Heidinger, Daniel J Dutton, Valerie Tarasuk, Norman R Campbell

**Affiliations:** 1 Department of Community Health Sciences, Faculty of Medicine, University of Calgary, 3rd Floor, TRW Building, 3280 Hospital Dr. NW, Calgary, Alberta T2N 4Z6, Canada; 2 Department of Nutritional Sciences, Faculty of Medicine, University of Toronto, 150 College Street, Toronto, Ontario M5S 3E2, Canada; 3 Departments of Medicine; Community Health Sciences; Physiology and Pharmacology, Faculty of Medicine, University of Calgary, Foothills Medical Centre, North Tower, 9th Floor, 1403 29th St. NW, Calgary, Alberta T2N 2 T9, Canada

**Keywords:** Sodium chloride, Dietary, Socio-economic factors, Population, Canada

## Abstract

**Introduction:**

In many countries including Canada, excess consumption of dietary sodium is common, and this has adverse implications for population health. Socio-economic inequities in sodium consumption seem likely, but research is limited. Knowledge of socio-economic inequities in sodium consumption is important for informing population-level sodium reduction strategies, to ensure that they are both impactful and equitable.

**Methods:**

We examined the association between socio-economic indicators (income and education) and sodium, using two outcome variables: 1) sodium consumption in mg/day, and 2) reported use of table salt, in two national surveys: the 1970/72 Nutrition Canada Survey and the 2004 Canadian Community Health Survey, Cycle 2.2. This permitted us to explore whether there were any changes in socio-economic patterning in dietary sodium during a time period characterized by modest, information-based national sodium reduction efforts, as well as to provide baseline information against which to examine the impact (equitable or not) of future sodium reduction strategies in Canada.

**Results:**

There was no evidence of a socio-economic inequity in sodium consumption (mg/day) in 2004. In fact findings pointed to a positive association in women, whereby women of higher education consumed more sodium than women of lower education in 2004. For men, income was positively associated with reported use of table salt in 1970/72, but negatively associated in 2004.

**Conclusions:**

An emerging inequity in reported use of table salt among men could reflect the modest, information-based sodium reduction efforts that were implemented during the time frame considered. However, for sodium consumption in mg/day, we found no evidence of a contemporary inequity, and in fact observed the opposite effect among women. Our findings could reflect data limitations, or they could signal that sodium differs from some other nutrients in terms of its socio-economic patterning, perhaps reflecting very high prevalence of excess consumption. It is possible that socio-economic inequities in sodium consumption will emerge as excess consumption declines, consistent with fundamental cause theory. It is important that national sodium reduction strategies are both impactful and equitable.

## Introduction

Excess dietary sodium consumption is a significant concern in many countries, due to its common occurrence and health implications. For example, national data from France, Finland, Canada, the USA, the UK, Brazil, and Turkey [1] show that average daily sodium consumption in these populations all exceed, some by a large margin, both the 2,000 mg/day maximum recommended by the World Health Organization [2], and the 2,300 mg/day Tolerable Upper Intake Level (UL) previously recommended by the U.S. Institute of Medicine [3]. In Canada, over 85% of men and over 60% of women age 19–70 have sodium intakes exceeding the 2,300 mg/day UL [4]. Most (75-80%) of this sodium comes from processed foods [5]. There is strong evidence that excess sodium intake is a risk factor for hypertension, stroke, and cardiovascular disease [6,7]. High blood pressure, which is directly linked with high sodium intake, is considered the leading preventable risk factor for death in the world [8].

In addition to high prevalence, socio-economic inequities in health problems are a prominent concern [9]. Socio-economic inequities in health are differences in health along social and economic axes, which are problematic to the extent that they are avoidable [10,11]. The dynamics of global capitalism since the 1970s have produced a widening gap between rich and poor [12,13]. This growing gap has implications for population health: large, persistent, and in many cases increasing socio-economic inequities have been documented for many health indicators, including those linked to high sodium intake such as heart disease and hypertension [14,15].

Socio-economic inequities exist for various aspects of diet. For example, Tarasuk et al. [16] observed that higher income and/or education was associated with a better nutrition profile, including: greater intake of fruit and vegetables, vitamins, minerals, and fibre [sodium was not examined]. Socio-economic inequities in diet reflect a number of factors, including food affordability, availability, preferences, food meaning and symbolism, and social and cultural norms [17]. Global trends related to agricultural production and trade, food processing and retailing, and advertizing and promotion have contributed to lower quality (i.e., highly processed) foods being highly accessible to those of lower socio-economic circumstances [18]. Because sodium serves a myriad of functions in processed foods, and because of the high accessibility of processed foods to those of lower socio-economic circumstances [18], it is plausible that sodium consumption is higher among those of lower socio-economic position [19]. However, direct evidence is limited.

Two Canadian population-based studies reported positive associations between socio-economic factors and sodium consumption based on food purchase [20] and intake based on a 24 hour dietary recall [21]. However, the association was reduced to non-significance when adjusting for energy differences in one study [20], suggesting that higher sodium among higher income people reflects that they purchase more food. Internationally, British national data [22] indicated an inverse association between socio-economic position and sodium intake (diet survey and 24-hour urine); Finnish data (1979–2002) revealed an inverse association between education and sodium (from 24-hour urine samples), in certain provinces [23]; U.S. data (from 2003/04 NHANES) showed a positive association between income/education and sodium (dietary interview) [24]; and a Swiss study [25] revealed no association between dietary salt intake (FFQ) and either occupation or education among adults in Geneva.

In recognition of the high prevalence of excess sodium consumption and the associated health implications, a growing number of countries are developing national sodium reduction strategies [26]. In general, these strategies align with the population-level approach to prevention as articulated by Rose [27], in that they target whole populations, without regard to variation in individual risk status, and thus have the potential to impact health at the population level. Reduction of dietary sodium lends itself to a population-level intervention approach for several reasons: first, as noted, in many countries a majority consumes excess sodium, and thus sodium reduction is broadly pertinent. Second, the association between sodium intake and blood pressure is linear with no obvious threshold [1], and thus there is no clear cutoff with which to identify a discrete high-risk group of individuals for targeted intervention. Third, several modeling studies have illustrated that the population-level impact of a widespread reduction (even a small reduction) in sodium intake is potentially very large, and would have significant cost savings [28,29].

The specific strategies included in these national initiatives vary [30] and may range in character from those that are more agentic (i.e., efforts to encourage behaviour change among individuals, such as by educating the public about the health implications of excess sodium consumption) to those that are more structural (i.e., efforts to modify the conditions in which behaviors occur, such as by working with food companies to set targets for maximum levels of sodium in food products) [31,32]. There is concern, which is backed up empirically, that intervention strategies of a more agentic nature could, in the absence of accompanying structural approaches, contribute to a widening or worsening of socio-economic inequities in health outcomes [31,33-36]. This in part reflects socio-economic variation in ability to benefit from those interventions, which may require active uptake. In Canada, during the time period studied in this research, sodium reduction efforts were modest and the main one was agentic in nature: in 1982, a moderation statement was added to Canada’s Food Guide, which encouraged Canadians to limit the amount of salt (along with sugar and fat) when selecting and preparing foods [37].

In summary, socio-economic inequities in sodium consumption seem likely, but research is limited in quantity and its results are mixed. Knowledge of socio-economic inequities in sodium consumption is important for the growing number of countries that are developing national sodium reduction strategies, to ensure that those strategies are equitable in their impact. The purpose of our study was to examine the association between socio-economic indicators (income and education) and sodium consumption (sodium consumption in mg/day, and reported use of table salt) among Canadian adults, in 1970/72 and 2004. This permitted us to explore whether there were any changes in socio-economic patterning in dietary sodium during a time characterized by modest, information-based (agentic) sodium reduction efforts, as well as to provide baseline information against which to examine the impact (equitable or not) of future sodium reduction strategies in Canada.

## Methods

### Data sources

We analyzed data from the 1970–72 Nutrition Canada Survey (NCS) and the 2004 Canadian Community Health Survey, Cycle 2.2 (CCHS). The surveys are not identical (see below), but have important similarities; both surveys: 1) used a stratified, multistage probability sampling technique to ensure representation of the ten provinces at the time; 2) included a sampling weight to account for the complex sampling design and patterns of non-response; 3) standardized the collection, interpretation, recording of data [38,39]; and 4) included a 24-hour dietary recall, and are to date the only Canadian national surveys to have done so (there are no more recent national dietary data for the Canadian population).

The 1970–72 NCS was administered by the Food and Drug Directorate of Health and Welfare Canada [40]. The target population was residents (all ages) of the ten provinces, excluding “Indians in bands and persons living in institutions and military camps” [38] and the sampling frame was a list of households based on the Canadian census. The response rate was 46% [38]. Data were collected via interview and during a clinic visit. The 2004 CCHS was Canada’s second national nutrition survey. The target population was Canadians age 2 years and older living in the ten provinces, excluding those living on First Nation reserves, in institutions, in prisons, in some remote areas, and who were regular members of the Canadian Armed Forces [39]. The sample was drawn using two sampling frames: an area frame designed for the Canadian Labour Force Survey which covers nearly the entire population, and a list frame created using household information of respondents to a previous iteration of the CCHS (cycle 2.1). The response rate was 76.5% [39]. We focused on men and women of working age (25–64 years) living in the ten provinces.

### Variables

Sodium consumption was measured in two ways: Sodium in milligrams per day was computed from the 24-hour dietary recall data from each survey. In the 1970–72 NCS, we merged recall data with data from the survey’s nutrient file, which was based on a U.S. food composition database [41] supplemented by several Canadian food codes for cookies and cakes [42]. In the 2004 CCHS, nutrients were estimated based on the Canadian Nutrient File (CNF) version 2001b supplement, a recipe file, and survey foods (food items that were not in the CNF but for which some nutritional information was available [39]). The CNF 2001b supplement is based on the USDA National Nutrient Database for Standard Reference (up to and including SR17); modifications include levels of fortification and regulatory standards specific to Canada, some Canadian only foods, and some brand name foods [39]). Reported use of table salt was based on a single item in the 1970–72 NCS: “Do you use free-flowing table salt?” (yes/no) and a composite variable in the 2004 CCHS: “How often is salt added at the table/while cooking?” which we dichotomized into yes (very often, occasionally, rarely) and no (never).

Predictor variables were income and education. For income, the 1970–72 NCS variable was “total annual family income from all sources”, which “includes family allowance, family assistance, old age benefits, welfare payments [and] should also include an estimate of the value of wages paid in kind, e.g., milk, fuel, staples” [42]. To simplify the models, the original 6-category variable was collapsed into three categories: $3,999 and less, $4,000-$9,999, and $10,000 and over^a^. For the 2004 CCHS we used the income adequacy variable which is derived from several questions about amount and sources of (gross) income and household size. Guided by the distribution of the data we created a three-category variable: lowest and lower middle; upper-middle; and highest income adequacy. While these dollar values are not directly comparable over time, the categories permit us to examine the relative differences between those with more, versus less, income in the population at the time.

Education in the 1970–72 NCS was recorded as a semi-continuous variable indicating the highest grade achieved (range 1–13); the number of years after high school, other than college, successfully achieved (range 1–9); or the number of years of college successfully completed (range 1–9). Credentials (e.g., high school graduation) were not recorded. Guided by the distribution of the data and a desire to have somewhat meaningful categories, we created a four category variable: grade 8 completed or less; grade 9–13 completed; some (range 1–9 years) post-high school other than college completed; and some (range 1–9 years) college completed. For the 2004 CCHS we used a four-category variable based on credentials: less than secondary graduation; secondary graduation +/− some (incomplete) post-secondary; credential below bachelor’s degree (e.g., certificate); and bachelor’s degree or above. Again, though categories are not directly comparable, they permit us examine the relative differences between those with more, versus less, education in the population at the time.

We adjusted for potential confounders: age, region of residence (British Columbia, Prairie provinces, Ontario, Quebec, Atlantic provinces), urban/rural residence, employment status (employed or not), marital status (married or common law versus not; CCHS only; not available in NCS), and birthplace (in the ten Canadian provinces versus elsewhere for the NCS; in Canada or elsewhere for the CCHS). We also adjusted for energy intake (kcal) estimated from the 24 hour recall, recognizing the correlation between sodium and energy intakes which could introduce error (higher sodium intake could be an artifact of higher reported total food intake; lower sodium intakes could be a function of under-reporting or lower reported dietary intakes).

### Analysis

First, we regressed sodium (mg/day) on income and education, unadjusted and adjusted for covariates, using ordinary least squares (OLS) regression. Second, to complement the OLS analysis which yields average effects of income and education, we computed the concentration index of inequality [43] which indicates how concentrated the health outcome (sodium, mg/day) is along the distribution income and education, taking into account the different proportions in the categories at different time periods. Third, we regressed reported use of table salt (yes/no) on income and education, unadjusted and adjusted for covariates using binary logistic regression.

In the CCHS, the 24-hour dietary recall was completed a second time for a sub-sample of respondents, which permits application of a correction algorithm for estimating usual intake. We used the algorithm produced by the National Cancer Institute (NCI) which, like other modeling methods for estimating usual intake of nutrients, uses information gleaned from the second recalls to analytically estimate and ‘remove’ the effects of within-person variation from the entire observed distribution of intakes [44]. To explore whether our OLS findings are robust to the usual intake estimation, we estimated distributions of usual sodium intake for all sex-SES groups, and compared 10^th^, 25^th^, 50^th^, 75^th^, and 90^th^ percentile estimates, and the mean, between SES groups using independent samples t-test.

We used Stata (v. 11.2), and SAS (v. 9.2) software. All models incorporate sampling weights as directed by Statistics Canada. For OLS and binary logistic models, confidence intervals were computed using standard Stata procedures; the reason being that the 1970/72 NCS does not come with bootstrap weights and our aim in those analyses was to maximize comparability of datasets. However, the standard errors associated with the percentiles of the estimated distribution of typical sodium consumption from the 2004 CCHS were estimated by a resampling method that is written within the NCI SAS macros, using the Statistics Canada bootstrapping weights. We ran models separately for men and women. The Conjoint Health Research Ethics Board at the University of Calgary approved the study (ID # E-24264).

## Results

Descriptive statistics are shown in Table 1. A total of 74 (1.6%) and 1,761 (14.4%) respondents were excluded due to missing data on one or more variables in the NCS and CCHS respectively. In the NCS, those with complete data consumed more energy (kcal), were older, were more likely to have lower educational attainment, and were more likely to be employed than those with incomplete data (p < .05). In the CCHS, most missing data occurred for income (n = 916) and for country of birth (n = 612). Those with complete data in the CCHS consumed more energy (kcal) and sodium; were more likely to be in the highest income category, to live in Quebec or the Atlantic region, and to be employed; and less likely to live in an urban environment, compared to those with incomplete data (p < .05).

**Table 1:** Weighted descriptive statistics (mean, SD or %) by survey, stratified by sex

**Variable**		**1970-72 NCS**		**2004 CCHS**	
		**Males (n = 1,974)**	**Females (n = 2,566)**	**Males (n = 4,837)**	**Females (n = 5,612)**
Outcome variables					
Sodium intake, mg/day		2880.8 (64.6)	1967.5 (44.9)	3223.3 (18.3)	2913.6 (14.1)
Use of table salt	Yes	97.7%	98.5%	92.8%	92.9%
	No	2.3%	1.5%	7.2%	7.1%
Predictor variables					
Income^1^	Low	13.5%	16.7%	19.4%	26.1%
	Middle	57.4%	52.1%	36.6%	35.2%
	High	29.0%	31.2%	44.0%	38.7%
Education^2^	Lowest	35.2%	32.0%	15.9%	13.8%
	Low-mid	42.6%	45.4%	23.2%	25.6%
	Mid-high	8.2%	14.9%	38.1%	36.7%
	Highest	14.0%	7.7%	22.8%	23.8%
Covariates					
Age		42.9 (.51)	42.5 (.43)	44.0 (.26)	43.5 (.24)
Energy intake (kcal)		2867.8 (49.2)	1822.7 (27.9)	2413.1 (26.4)	1827.0 (19.2)
Region	B.C.	12.4%	10.7%	12.3%	13.4%
	Prairies	17.8%	15.6%	17.1%	16.0%
	Ontario	32.3%	36.2%	37.2%	37.8%
	Quebec	28.4%	29.2%	25.0%	24.5%
	Atlantic	9.0%	8.3%	8.4%	8.3%
Urban/rural	Urban	72.0%	74.8%	80.2%	81.7%
	Rural	28.0%	25.2%	19.8%	18.3%
Birthplace^3^	Canada	73.2%	80.5%	83.5%	84.4%
	Other	26.8%	19.5%	16.5%	15.6%
Employment status	Employed	87.5%	29.6%	90.0%	79.6%
	Not employed	12.5%	70.4%	10.0%	20.4%
Marital status	Married or common law	N/A	N/A	74.1%	72.4%
	Other			25.9%	27.6%

Notes:

^1^Income in the NCS: low (<=$3,999), middle ($4,000-$9,999), high (> = $10,000). Income in the CCHS refers to income adequacy based on gross household income and # of people in household: low (lowest and lower middle $ adequacy); middle (upper-middle $ adequacy); high (highest $ adequacy).

^2^Education in the NCS: low (grade 8 or less); low-middle (grade 9–13); middle-high (some [1–9 years] post high school other than college); high (some [range 1–9 years] college).

^3^Birthplace: in the ten Canadian provinces versus elsewhere (NCS); in Canada or elsewhere (CCHS).

Results from the OLS regressions are shown in Tables 2 and 3. For males (Table 2), neither income nor education was associated with sodium intake (mg/day), in the 1970–72 NCS or in the 2004 CCHS. For females (Table 3), there was no effect of income in either dataset. For education, there were significant but opposite effects for women in 1970/72 and 2004: in 1970/72, women with the highest education (and women in the lower-middle education category, fully adjusted model only) consumed significantly less sodium than women with the lowest education. In 2004, women in the highest (at p<.10) and second highest education categories consumed significantly more sodium than women in the lowest education category, though this effect was not observed in the fully adjusted model.

**Table 2:** Results of OLS regression analyses for MALES in a) the NCS 1970–72 (n = 1,974) and b) the CCHS 2004 (n = 4,837), with sodium intake (mg/day) regressed on socio-economic variables and covariates

**Predictor variable**		**NCS 1970-72**			**CCHS 2004**		
		**Unadjusted estimates**^**1 **^**coefficient (95% CI)**	**Partially adjusted model**^**2 **^**coefficient (95% CI)**	**Fully adjusted model**^**3 **^**coefficient (95% CI)**	**Unadjusted estimates**^**1 **^**coefficient (95% CI)**	**Partially adjusted model**^**2 **^**coefficient (95% CI)**	**Fully adjusted model**^**3 **^**coefficient (95% CI)**
Income (ref: low)	Middle	116.4 (−256.4 to 489.3)	−27.3 (−460.2 to 405.6)	−67.1 (−390.9 to 256.8)	172.3 (−147.6 to 492.2)	100.6 (−232.9 to 434.0)	6.8 (−200.2 to 213.7)
	High	−110.3 (−503.9 to 293.3)	−205.2 (−632.1 to 221.7)	−301.8 (−667.8 to 64.3)	69.5 (−251.5 to 390.5)	45.7 (−305.4 to 396.8)	−17.4 (−243.7 to 209.0)
Education (ref: lowest)	Low-mid	95.4 (−195.1 to 386.0)	56.9 (−232.2 to 345.9)	5.5 (−233.9 to 245.0)	52.7 (−270.1 to 375.5)	−31.1 (−366.3 to 304.1)	82.8 (−154.0 to 319.7)
	Mid-high	11.4 (−409.0 to 431.7)	−14.6 (−411.4 to 382.2)	−119.4 (−469.5 to 230.8)	42.1 (−265.4 to 349.5)	−81.1 (−406.2 to 243.9)	2.2 (−233.4 to 237.8)
	Highest	−257.7 (−647.4 to 131.9)	−158.3 (−575.7 to 259.2)	−49.7 (−479.1 to 379.7)	147.6 (−457.1 to 161.9)	−238.3 (−585.7 to 109.2)	−141.2 (−408.5 to 126.1)
Energy intake (kcal)		.73 (.57 to .89)**	--	.73 (.58 to .88)**	1.28 (1.2 to 1.4)**	--	1.3 (1.2 to 1.4)**
Age		−16.1 (−26.3 to −5.9)**	−15.8 (−26.6 to −4.9)**	2.6 (−6.1 to 11.2)	−11.8 (−19.5 to −4.1)**	−12.4 (−20.9 to −3.9)**	3.7 (−2.4 to 9.7)
Region (ref: B.C.)	Prairies	−56.2 (−424.0 to 311.5)	−172.7 (−543.4 to 198.1)	−305.1 (−602.5 to −7.8)*	−224.1 (−592.7 to 144.5)	−289.6 (−654.5 to 75.4)	32.9 (−239.5 to 305.3)
	Ontario	−230.2 (−631.2 to 170.8)	−243.8 (−632.4 to 144.9)	−253.3 (−578.7 to 72.2)	−518.0 (−870.5 to −165.5)**	−539.5 (−885.9 to −193.1)**	−213.8 (−468.0 to 40.4)†
	Quebec	141.7 (−197.3 to 480.7)	51.6 (−306.4 to 409.6)	−56.5 (−366.5 to 253.5)	47.4 (−349.7 to 444.5)	35.0 (−376.1 to 446.1)	125.9 (−158.5 to 410.3)
	Atlantic	94.3 (−255.3 to 443.9)	−74.4 (−454.6 to 305.8)	−159.9 (−455.5 to 135.8)	−364.8 (−719.4 to −10.2)*	−453.5 (−823.4 to −83.6)*	−188.0 (−468.6 to 92.6)
Urban residence (ref: rural)		−337.2 (−611.8 to −62.6)*	−296.0 (−575.4 to −16.7)*	−262.8 (−482.2 to −43.5)*	127.2 (−83.3 to 337.7)	87.2 (−124.6 to 299.0)	.33 (−153.4 to 154.1)
Born outside of Canada (ref: born in Canada)^4^		343.9 (40.1 to 647.7)*	214.5 (−109.2 to 538.2)	185.4 (−110.3 to 481.1)	246.6 (−83.2 to 576.4)	138.9 (−207.5 to 485.2)	66.6 (−149.7 to 282.9)
Employed (ref: not employed)		386.5 (−155.6 to 928.6)	345.9 (−268.4 to 960.2)	321.9 (−234.3 to 882.1)	387.4 (130.9 to 643.8)**	323.9 (20.8 to 627.1)*	52.6 (−134.5 to 239.7)
Marital status (ref: not married or common-law)		N/A	N/A	N/A	124.4 (−73.8 to 322.6)	205.5 (3.7 to 407.3)*	79.3 (−66.1 to 224.8)

Notes.

CCHS 2004 coefficients are based on the non-NCI-corrected data, to increase comparability with the 1970–72 NCS.

^1^Column contains bi-variate associations between each predictor variable and the outcome variable (sodium intake in mg/day).

^2^Column contains associations from single model containing all variables except energy intake (kcal).

^3^Column contains associations from single model containing all variables.

^4^Birthplace: in the ten Canadian provinces versus elsewhere (NCS); in Canada or elsewhere (CCHS).

**p < .01; *p < .05; †p < .10.

**Table 3:** Results of OLS regression analyses for FEMALES in a) the NCS 1970–72 (n = 2,566) and b) the CCHS 2004 (n = 5,612), with sodium intake (mg/day) regressed on socio-economic variables and covariates

**Predictor variable**		**NCS 1970-72**			**CCHS 2004**		
		**Unadjusted estimates**^**1 **^**coefficient (95% CI)**	**Partially adjusted model**^**2 **^**coefficient (95% CI)**	**Fully adjusted model**^**3 **^**coefficient (95% CI)**	**Unadjusted estimates**^**1 **^**coefficient (95% CI)**	**Partially adjusted model**^**2 **^**coefficient (95% CI)**	**Fully adjusted model**^**3 **^**coefficient (95% CI)**
Income (ref: low)	Middle	152.2 (−35.0 to 339.5)	166.9 (−39.2 to 373.0)	68.8 (−103.2 to 240.8)	83.7 (−94.8 to 262.2)	18.0 (−168.8 to 204.8)	−5.6 (−152.0 to 140.7)
	High	−2.3 (−226.7 to 222.0)	102.7 (−133.1 to 338.5)	−46.3 (−257.4 to 164.8)	116.2 (−51.8 to 284.1)	54.9 (−140.9 to 250.7)	54.5 (−105.0 to 214.0)
Education (ref: lowest)	Low-mid	−147.9 (−354.0 to 58.3)	−173.6 (−380.3 to 33.2)	−216.7 (−406.6 to −26.7)*	138.3 (−63.6 to 340.1)	151.8 (−58.5 to 362.1)	−15.5 (−191.4 to 160.4)
	Mid-high	52.3 (−245.6 to 350.2)	91.9 (−224.4 to 408.3)	−16.7 (−286.4 to 253.0)	383.5 (169.9 to 597.0)**	352.5 (129.0 to 576.1)**	106.0 (−80.5 to 292.5)
	Highest	−350.3 (−671.7 to −28.9)*	−362.3 (−709.3 to −15.3)*	−473.2 (−795.4 to −151.0)**	244.7 (28.2 to 461.1)*	223.3 (−16.7 to 463.3)†	−90.5 (−299.6 to 118.6)
Energy intake (kcal)		.81 (.70 to .92)**	--	.81 (.71 to .92)**	1.3 (1.2 to 1.3)**	--	1.3 (1.2 to 1.3)**
Age		−8.7 (−16.9 to -.53)*	−9.8 (−17.5 to −1.99)*	-.91 (−7.5 to 5.7)	−9.3 (−15.5 to −3.1)**	−8.0 (−14.4 to −1.6)*	2.1 (−2.7 to 6.9)
Region (ref: B.C.)	Prairies	174.1 (−19.5 to 367.8)†	183.1 (−21.9 to 388.1)†	133.1 (−31.6 to 297.8)	−75.5 (−293.6 to 142.5)	−89.6 (−312.6 to 133.4)	−13.3 (−181.5 to 154.9)
	Ontario	117.9 (−105.2 to 341.0)	79.4 (−143.0 to 301.8)	22.2 (−161.5 to 206.0)	−207.5 (−424.5 to 9.6)†	−215.3 (−430.0 to -.65)*	−45.4 (−201.9 to 111.1)
	Quebec	430.2 (251.7 to 608.7)**	397.7 (207.9 to 587.4)**	137.7 (−32.2 to 307.6)	201.6 (−55.3 to 458.6)	265.5 (−7.4 to 538.3)†	72.5 (−117.6 to 262.5)
	Atlantic	132.8 (−36.3 to 301.9)	119.5 (−71.7 to 310.8)	54.7 (−104.5 to 213.8)	−55.2 (−290.4 to 180.1)	−43.6 (−294.5 to 207.3)	70.8 (−111.7 to 253.3)
Urban residence (ref: rural)		16.8 (−136.3 to 169.8)	20.2 (−137.3 to 177.7)	31.2 (−96.4 to 158.7)	147.3 (−22.3 to 316.9)†	135.7 (−40.1 to 311.5)	113.6 (−22.1 to 249.4)
Born outside of Canada (ref: born in Canada)^4^		71.6 (−200.4 to 343.7)	−2.2 (−267.2 to 262.8)	−46.1 (−285.1 to 192.9)	48.8 (−189.1 to 286.7)	−48.1 (−288.9 to 192.7)	−38.6 (−218.3 to 141.1)
Employed (ref: not employed)		17.4 (−187.5 to 222.3)	30.8 (−167.0 to 228.5)	−14.0 (−182.5 to 154.4)	177.5 (25.1 to 330.0)*	90.9 (−67.8 to 249.6)	135.3 (3.0 to 267.7)*
Marital status (ref: not married or common-law)		N/A	N/A	N/A	175.1 (30.5 to 319.7)*	170.6 (18.2 to 323.0)*	9.6 (−112.5 to 131.6)

Notes.

CCHS 2004 coefficients are based on the non-NCI-corrected data, to increase comparability with the 1970–72 NCS.

^1^Column contains bi-variate associations between each predictor variable and the outcome variable (sodium intake in mg/day).

^2^Column contains associations from single model containing all variables except energy intake (kcal).

^3^Column contains associations from single model containing all variables.

^4^Birthplace: in the ten Canadian provinces versus elsewhere (NCS); in Canada or elsewhere (CCHS).

**p < .01; *p < .05; †p < .10.

Results from the comparison of estimated usual intake distribution across income and education groups, at the 10^th^, 25^th^, 50^th^, 75^th^, and 90^th^ percentiles and the mean, are shown in Table 4. There was only one significant difference observed: the mean estimated usual sodium intake for women in the second highest education category (mid-high) was significantly higher than that for women in the lowest education category (t = 2.6, p < .05).

**Table 4:** Estimates of usual intake of sodium in mg/day (mean, 95% confidence interval) among men (n = 4,837) and women (n = 5,612) from the CCHS, using the NCI method

**Men**							
		**Mean (95% CI)**	**10**^**th **^**percentile mean (95% CI)**	**25**^**th **^**percentile mean (95% CI)**	**50**^**th **^**percentile mean (95% CI)**	**75**^**th **^**percentile mean (95% CI)**	**90**^**th **^**percentile mean (95% CI)**
Income	Lowest	3764.2 (2896.4 to 4631.95)	2267.3 (782.7 to 3751.9)	2848.7 (1950.2 to 3747.2)	3605.5 (2914.9 to 4296.2)	4524.7 (2852.9 to 6196.6)	5472.8 (2545.4 to 8400.3)
	Middle	3544.3 (3332.1 to 3756.6)	2088.1 (662.5 to 3513.7)	2675.4 (1756.0 to 3594.9)	3426.8 (3147.0 to 3706.6)	4300.7 (3534.4 to 5066.9)	5165.1 (3495.3 to 6834.8)
	Highest	3509.8 (3296.5 to 3723.1)	2039.5 (1302.6 to 2776.3)	2628.7 (2145.7 to 3111.8)	3393.8 (3185.4 to 3602.2)	4255.1 (3752.9 to 4757.3)	5131.9 (4122.0 to 6141.9)
Education	Lowest	3532.7 (3151.6 to 3913.9)	2616.1 (1415.2 to 3816.9)	2998.8 (2278.5 to 3719.1)	3481.6 (3124.5 to 3838.7)	4009.1 (3115.1 to 4903.2)	4520.0 (2861.5 to 6178.4)
	Low-mid	3614.0 (3335.3 to 3892.7)	2688.3 (1371.8 to 4004.8)	3085.9 (2288.9 to 3882.9)	3564.4 (3266.5 to 3862.5)	4090.0 (3371.3 to 4808.6)	4606.2 (3175.2 to 6037.2)
	Mid-high	3650.3 (3404.2 to 3896.4)	2508.1 (926.7 to 4089.4)	2975.6 (2012.7 to 3938.5)	3570.0 (3298.6 to 3841.3)	4234.7 (3373.1 to 5096.3)	4905.1 (3098.0 to 6712.1)
	Highest	3515.8 (3217.2 to 3814.5)	2141.8 (733.2 to 3550.4)	2694.1 (1805.1 to 3583.0)	3419.0 (3114.6 to 3723.4)	4213.9 (3371.7 to 5056.1)	5014.0 (3302.2 to 6725.7)
**Women**							
		**Mean (95% CI)**	**10**^**th**^** percentile mean (95% CI)**	**25**^**th**^** percentile mean (95% CI)**	**50**^**th**^** percentile mean (95% CI)**	**75**^**th**^** percentile mean (95% CI)**	**90**^**th **^**percentile mean (95% CI)**
Income	Lowest	2692.0 (2482.8 to 2901.2)	1571.4 (1148.7 to 1994.2)	2011.7 (1705.9 to 2317.4)	2594.6 (2384.0 to 2805.3)	3261.1 (2904.0 to 3618.3)	3935.5 (3305.8 to 4565.3)
	Middle	2689.4 (2402.3 to 2976.5)	2003.8 (1130.3 to 2877.2)	2299.2 (1729.5 to 2868.8)	2655.0 (2372.7 to 2937.4)	3037.1 (2566.2 to 3508.1)	3419.2 (2517.4 to 4321.1)
	Highest	2662.6 (2391.2 to 2934.0)	2110.3 (1486.9 to 2733.8)	2347.2 (1950.5 to 2743.9)	2635.2 (2378.0 to 2892.4)	2951.7 (2464.3 to 3439.1)	3252.2 (2394.9 to 4109.5)
Education	Lowest	2466.8 (2241.7 to 2691.8)	2463.4 (763.8 to 4163.1)	2465.1 (1428.4 to 3501.8)	2466.8 (2211.8 to 2721.8)	2468.5 (1532.1 to 3404.9)	2470.1 (481.5 to 4458.6)
	Low-mid	2632.3 (2489.7 to 2774.9)	1945.2 (1277.7 to 2612.8)	2235.1 (1821.4 to 2648.9)	2591.7 (2439.9 to 2743.5)	2988.7 (2611.5 to 3365.9)	3375.0 (2615.7 to 4134.4)
	Mid-high	2860.8 (2671.2 to 3050.4)*	1874.8 (556.5 to 3193.1)	2278.8 (1464.0 to 3093.7)	2791.0 (2561.3 to 3020.7)	3365.2 (2678.2 to 4052.2)	3928.0 (2488.5 to 5367.5)
	Highest	2676.3 (2370.9 to 2981.8)	1820.7 (865.3 to 2776.0)	2173.9 (1507.0 to 2840.9)	2622.8 (2288.1 to 2957.6)	3113.9 (2717.9 to 3509.8)	3592.9 (2776.3 to 4409.5)

*significantly different from low education at p < .05, based on independent samples t-test.

The concentration index can range from −1 to +1, with negative values (if statistically different from zero) indicating disproportionate concentration of the health problem within lower socio-economic groups. The concentration indices did not differ significantly from zero for men or women in 1970/72, or for men in 2004. For women in 2004, the concentration index for education-related inequality was statistically significant and positive, though small (0.007 [95% confidence interval 0.002 to 0.012], p = .011).

Results of binary logistic regression are shown in Tables 5 and 6. For men (Table 5), in 1970/72 those of middle income had higher odds of reporting table salt use than those of lowest income (at p < .10 in the partially and fully adjusted models); whereas in 2004 those of highest income had lower odds of reporting table salt use than those of lowest income. For women (Table 6), no effects were observed at the p < .05 level (though there was a marginally significant negative effect of high income in 2004, similar to that observed in men).

**Table 5:** Results of binary logistic regression analyses for MALES in a) the NCS (n = 1,974) and b) the CCHS (n = 4,837), with reported use of table salt (yes versus no) regressed on socio-economic variables and covariates

**Predictor variable**		**NCS 1970-72**			**CCHS 2004**		
		**Unadjusted estimates**^**1 **^**odds ratio (95% CI)**	**Partially adjusted model**^**2 **^**odds ratio (95% CI)**	**Fully adjusted model**^**3 **^**odds ratio (95% CI)**	**Unadjusted estimates**^**1 **^**odds ratio (95% CI)**	**Partially adjusted model**^**2 **^**odds ratio (95% CI)**	**Fully adjusted model**^**3 **^**odds ratio (95% CI)**
Income (ref: low)	Middle	3.6 (1.3 to 9.6)*	3.30 (.90 to 12.1)†	3.6 (.93 to 14.1)†	1.4 (.86 to 2.1)	1.09 (.69 to 1.7)	1.08 (.68 to 1.7)
	High	3.1 (.91 to 10.7)†	2.8 (.55 to 13.8)	2.96 (.53 to 16.4)	.67 (.45 to 1.02)†	.50 (.31 to .80)**	.49 (.31 to .79)**
Education (ref: lowest)	Low-mid	1.5 (.57 to 3.8)	.79 (026 to 2.4)	.69 (.23 to 2.1)	1.2 (.66 to 2.3)	1.2 (.60 to 2.2)	1.2 (.60 to 2.2)
	Mid-high	6.4 (.82 to 50.9)†	3.6 (.38 to 34.5)	3.2 (.33 to 30.7)	.76 (.44 to 1.3)	.68 (.38 to 1.2)	.69 (.38 to 1.2)
	Highest	1.9 (.38 to 9.6)	.76 (.12 to 5.0)	.58 (.09 to 3.9)	.86 (.47 to 1.6)	.88 (.46 to 1.7)	.89 (.46 to 1.7)
Energy intake (kcal)		.9996 (.9991 to1.00005)†	--	.9992 (.9987 to .9997)**	1.0002 (.99993 to 1.0004)	--	1.0001 (.99986 to 1.0003)
Age		.93 (.88 to .98)**	.93 (.88 to .99)*	.91 (.86 to .96)**	.99 (.97 to 1.004)	.99 (.97 to 1.01)	.99 (.97 to 1.008)
Region (ref: B.C.)	Prairies	2.3 (.50 to 11.1)	2.7 (.53 to 13.5)	3.2 (.56 to 18.6)	.69 (.36 to 1.3)	.72 (.38 to 1.4)	.73 (.38 to 1.4)
	Ontario	3.9 (.72 to 21.3)	3.7 (.74 to 18.1)	3.6 (.71 to 17.7)	.57 (.32 to .99)*	.57 (.32 to 1.0)†	.58 (.33 to 1.02)†
	Quebec	.86 (.27 to 2.7)	1.1 (.31 to 3.6)	1.1 (.31 to 3.9)	.79 (.40 to 1.5)	.85 (.42 to 1.7)	.86 (.43 to 1.74)
	Atlantic	3.8 (.95 to 15.4)†	4.5 (1.1 to 19.0)*	6.3 (1.3 to 30.3)*	.82 (.42 to 1.6)	.70 (.34 to 1.4)	.71 (.34 to 1.5)
Urban residence (ref: rural)		1.2 (.48 to 2.9)	1.0 (.43 to 2.4)	.98 (.42 to 2.3)	1.4 (.96 to 2.2)†	1.4 (.89 to 2.2)	1.4 (.88 to 2.2)
Born outside of Canada (ref: born in Canada)^4^		.32 (.09 to 1.2)†	.38 (.09 to 1.6)	.31 (.08 to 1.3)	.75 (.44 to 1.3)	.73 (.42 to 1.3)	.73 (.42 to 1.3)
Employed (ref: not employed)		.77 (.24 to 2.5)	.28 (.08 to .96)*	.33 (.09 to 1.2)†	1.96 (1.3 to 2.99)**	2.4 (1.5 to 3.8)**	2.4 (1.5 to 3.7)**
Marital status (ref: not married or common-law)		N/A	N/A	N/A	1.5 (1.07 to 2.2)*	1.8 (1.2 to 2.6)**	1.8 (1.2 to 2.6)**

Notes.

^1^Column contains bi-variate associations between each predictor variable and sodium intake.

^2^Column contains associations from single model containing all variables except energy intake (kcal).

^3^Column contains associations from single model containing all variables.

^4^Birthplace: in the ten Canadian provinces versus elsewhere (NCS); in Canada or elsewhere (CCHS).

**p < .01; *p < .05; †p < .10.

**Table 6:** Results of binary logistic regression analyses for FEMALES in a) the NCS (n = 2,566) and b) the CCHS (n = 5,612), with reported use of table salt (yes versus no) regressed on socio-economic variables and covariates

**Predictor variable**		**NCS 1970-72**			**CCHS 2004**		
		**Unadjusted estimates**^**1 **^**odds ratio (95% CI)**	**Partially adjusted model**^**2 **^**odds ratio (95% CI)**	**Fully adjusted model**^**3 **^**odds ratio (95% CI)**	**Unadjusted estimates**^**1 **^**odds ratio (95% CI)**	**Partially adjusted model**^**2 **^**odds ratio (95% CI)**	**Fully adjusted model**^**3 **^**odds ratio (95% CI)**
Income (ref: low)	Middle	.90 (.29 to 2.7)	.59 (.16 to 2.1)	.59 (.16 to 2.1)	.94 (.61 to 1.4)	.81 (.52 to 1.3)	.80 (.51 to 1.3)
	High	.93 (.23 to 3.8)	.45 (.13 to 1.6)	.45 (.13 to 1.6)	.74 (.47 to 1.2)	.64 (.40 to 1.03)†	.64 (.40 to 1.03)†
Education (ref: lowest)	Low-mid	.92 (.33 to 2.6)	.70 (.27 to 1.8)	.70 (.27 to 1.8)	1.2 (.69 to 2.2)	1.5 (.85 to 2.6)	1.4 (.82 to 2.5)
	Mid-high	.67 (.13 to 3.4)	.50 (.13 to 1.9)	.50 (.13 to 1.9)	1.2 (.71 to 2.1)	1.4 (.85 to 2.5)	1.4 (.81 to 2.4)
	Highest	4.8 (.81 to 28.2)†	4.5 (.66 to 30.8)	4.5 (.65 to 30.9)	.88 (.48 to 1.6)	1.1 (.59 to 2.1)	1.05 (.54 to 2.03)
Energy intake (kcal)		1.0001 (.9997 to 1.0005)	--	1.00002 (.9996 to 1.0005)	1.0003 (.99994 to 1.0006)	--	1.0003 (.99990 to 1.0006)
Age		.95 (.91 to .98)**	.94 (.90 to .98)**	.94 (.90 to .98)**	1.005 (.99 to 1.02)	1.01 (.99 to 1.02)	1.007 (.99 to 1.02)
Region (ref: B.C.)	Prairies	1.8 (.36 to 9.3)	2.1 (.33 to 13.6)	2.1 (.33 to 13.6)	.92 (.48 to 1.8)	1.02 (.54 to 1.9)	1.04 (.55 to 1.97)
	Ontario	4.7 (.78 to 28.8)†	5.5 (1.04 to 28.6)*	5.4 (1.03 to 28.8)*	.84 (.47 to 1.5)	.95 (.53 to 1.7)	.98 (.54 to 1.8)
	Quebec	1.6 (.56 to 4.6)	1.5 (.47 to 4.6)	1.5 (.44 to 4.9)	1.8 (.90 to 3.5)†	2.2 (1.2 to 4.2)*	2.2 (1.2 to 4.1)*
	Atlantic	36.1 (4.4 to 298.9)**	44.7 (5.4 to 372.5)**	44.7 (5.4 to 372.8)**	.69 (.38 to 1.3)	.81 (.44 to 1.5)	.83 (.44 to 1.5)
Urban residence (ref: rural)		1.3 (.52 to 3.01)	1.5 (.54 to 3.9)	1.5 (.54 to 3.9)	1.03 (.63 to 1.7)	1.05 (.61 to 1.8)	1.05 (.61 to 1.8)
Born outside of Canada (ref: born in Canada)^4^		.58 (.15 to 2.1)	.56 (.13 to 2.4)	.56 (.13 to 2.4)	.50 (.26 to .97)*	.46 (.23 to .91)*	.46 (.23 to .90)*
Employed (ref: not employed)		1.3 (.33 to 5.0)	1.2 (.39 to 3.8)	1.2 (.39 to 3.8)	1.1 (.75 to 1.7)	1.4 (.89 to 2.2)	1.4 (.90 to 2.2)
Marital status (ref: not married or common-law)		N/A	N/A	N/A	1.3 (.89 to 1.8)	1.5 (1.02 to 2.1)*	1.4 (1.0007 to 2.02)†

Notes.

^1^Column contains bi-variate associations between each predictor variable and sodium intake.

^2^Column contains associations from single model containing all variables except energy intake (kcal).

^3^Column contains associations from single model containing all variables.

^4^Birthplace: in the ten Canadian provinces versus elsewhere (NCS); in Canada or elsewhere (CCHS).

**p < .01; *p < .05; †p < .10.

## Discussion and conclusions

Main findings were as follows: (1) There was no evidence of an inequity in sodium consumption (mg/day) in 2004, for men or women (though there was a negative effect of high education in women in 1970/72). Though findings pointed to a positive effect in women whereby women of higher education consumed more sodium than women of lower education in 2004, the OLS effect did not hold in fully adjusted models and the concentration index was very small; thus we focus on absence of inequity for both men and women as the main finding. (2) For men, income was positively associated with reported use of table salt in 1970/72, but negatively associated in 2004.

The negative effect of income on reported use of table salt among men in 2004 (with a similar trend among women, though it did not reach statistical significance at the .05 level) is consistent with a contemporary inequity in reported use of table salt. This finding, which resembles socio-economic patterning observed for other reported health behaviors such as physical activity [45], likely reflects socio-economic patterning of awareness of, attendance to, concern about, and inclination and ability to engage in, health promoting activities. That the effect was positive in 1970/72 but negative in 2004 suggests that the inequity emerged between the time points. As noted in the introduction section, inequities in health (including health behaviours) can (unintentionally) emerge or increase in response to some health interventions; in particular, interventions of an agentic nature, whose impact is dependent on individual uptake which itself may depend on one’s social and economic resources [31,35]. Though national sodium reduction efforts were not prominent in Canada during the time frame studied, one initiative that did occur was the addition, in 1982, of a moderation statement to Canada’s Food Guide which encouraged Canadians to “select and prepare foods with limited amounts of fat, sugar, and salt” [37]. It is possible that uptake of this statement varied by income, thus contributing to the inequity observed here.

Though inequities in reported salt use are important, one must keep in mind that salt added at the table or while cooking constitutes only a small proportion of overall sodium intake (less than 10%) in most higher-income countries [5]. Thus, sodium consumption in mg/day is perhaps the more important metric in this study. For sodium consumption in mg/day, though there was an apparent educational inequity among women in 1970/72, there was no indication of inequity in 2004, and in fact for women findings pointed to a positive effect of education on sodium consumption. The absence of inequity in 2004 was somewhat contrary to expectations. One possible explanation is data limitations; for example, because nutrient estimates in the 2004 data were based on an older (i.e., 2001) nutrient file, they may not fully reflect the range of reduced-sodium food products. For example, a search of the Canadian Nutrient File [46] for reduced-sodium foods (i.e., “sodium-reduced”, “low sodium”, “low salt”, “salt-reduced”) revealed 21 items that were added to the database in 2001 or earlier, and 37 additional items that were added in 2004. Those 37 items were thus available on the market in 2004 but would not have been captured in the survey. To the extent that purchase of reduced-sodium foods is patterned by socio-economic status [47,48], those inequities could be masked. The international literature points to the possibility that study findings may depend on the method of sodium measurement: whereas studies that used a 24-hour urine sample (gold standard) showed socio-economic inequities in sodium consumption [22,23], others that used self-report methods showed either a positive association [24] or no association [25]). These differences could also reflect different dietary practices in those countries studied.

Data limitations notwithstanding, the absence of socio-economic inequities in sodium (mg/day) prompts consideration of the possibility that strong socio-economic inequities in sodium do not presently exist, perhaps reflecting the very high prevalence of excess intake [1,4]. With the ubiquity of sodium in the food environment [49], it may be that no socio-economic group is exempt. A similar pattern has been observed with obesity, where inequities have lessened as prevalence has increased [50,51]. An absence of socio-economic inequities in sodium would contrast with observations of inequities in some other aspects of diet [16]. One possible explanation pertains to trends in socio-economic patterning of eating outside of the home (e.g., in restaurants). Some studies [52] though not others [53] show a positive association between education and eating outside of the home, and sodium content of restaurant meals (including fast food and sit-down restaurants) has been found to be very high [54]. It is thus possible that more eating outside of the home is offsetting any nutritional advantages that higher education groups may otherwise have. If the observed absence of substantive and consistent socio-economic inequities in sodium consumption (mg/day) is accurate, and if it indeed reflects, in part, high prevalence of excess consumption, then it is important to consider that as population sodium intake decreases, inequities could emerge. This interpretation is consistent with fundamental cause theory (e.g., [55]) whereby as population health improves, inequities more or less always emerge due to emerging variation in people’s capacity to benefit from improvements. On the other hand, several studies have shown that population health improvements and worsening of socio-economic inequities do not necessarily co-occur. Drinking water fluoridation, mandatory food product fortification, and enforced seatbelt laws are examples of interventions for which improvements have occurred equitably [32,51,56,57].

From this point of view, it is important that national sodium reduction efforts include strategies that have been shown to be both impactful and equitable. In the UK, a national sodium reduction strategy has been implemented which includes sodium reduction in processed foods initiated voluntarily by companies, consumer education campaigns, front-of-package labeling, and restrictions on food marketing to children. Evidence is beginning to emerge that the UK strategy is both impactful and equitable [58,59]. In Canada, efforts were undertaken to devise a national strategy [1], which was in part modeled after the UK initiative. Unfortunately, progress on national sodium reduction in Canada appears to have stalled [60].

Our study has limitations. While the 24-hour dietary recall has practical advantages, it has limited reliability and validity [61], even when a second recall is available. The two surveys are not directly comparable, but they have important similarities which permit some insight to be drawn into social change (or lack thereof) over time. The response rate for the 1970/72 NCS was low (46%). However, crude comparison of the 1970/72 NCS with the 1971 Canadian census^b^ revealed reasonable similarity in terms of the proportion of women (48.7% vs 49.9%); those living in urban settings (73.3% vs 78.0%); and population by geographic region (8.5%, 28.4%, 35.3%, 16.4% vs 11.4% in the NCS; 9.6%, 28.0%, 35.8%, 16.5%, and 10.2% in Atlantic, Quebec, Ontario, Prairies, B.C. regions respectively). A final limitation is that we only focused on one axis of inequity: socio-economic inequity. Inequities in Canada take many other forms, including geography (e.g., neighbourhood, urban/rural, and larger geographic region) and Aboriginal status [62-64]. Sodium inequities along these other axes are important foci for future research studies.

In conclusion, the growing number of national dietary sodium reduction initiatives is a trend with potentially significant positive implications for population health. To ensure that these initiatives are both impactful and equitable, high-quality data (including 24-hour urine samples) to permit the monitoring of sodium consumption, including inequities in sodium consumption, is essential.

## Endnotes

^a^Although the income variable from the NCS did not take household size into account, the dataset does contain a variable indicating “number of residents in dwelling”. A one-way (number of residents [continuous] by total family income [high, medium, low]) analysis of variance (not shown) indicated that families in the lowest income category had significantly fewer residents than families in the middle and highest income categories, which would tend to bias effects towards the null. There were no other differences.

^b^Available on the Statistics Canada website under “Historical Statistics of Canada”. http://www.statcan.gc.ca/pub/11-516-x/sectiona/4147436-eng.htm.

## Abbreviations

CCHS: Canadian Community Health Survey (specifically Cycle 2.2, 2004); CNF: Canadian Nutrient File; NCI: National Cancer Institute; NCS: Nutrition Canada Survey (1970–72); NHANES: National Health and Nutrition Examination Survey (U.S.A.); OLS: Ordinary Least Squares (regression); UK: United Kingdom; UL: Tolerable Upper Intake Level (U.S. Institute of Medicine); USA: United States of America.

## Competing interests

Lindsay McLaren, Shayla Heidinger, Daniel J. Dutton, and Valerie Tarasuk have no conflicts of interest to declare. Norm Campbell discloses the following:

● → 2007: received travel funds from McCain to attend a regional Dieticians of Canada meeting to talk about the importance of reducing dietary sodium.

● → 2008: received samples of Mrs DASH; asked not to send more.

● → 2010: received travel funds from Boehringer Ingelheim to attend two hypertension meetings.

● → Receives salary from HSFC and CIHR to lead and coordinate efforts to prevent and control hypertension.

● → Is an unpaid consultant for many NGOs and GOs but none with a commercial flavor.

● → Receives occasional honoraria for speaking to academic groups on sodium (e.g. Canadian Council of Cardiovascular Nurses).

## Authors’ contributions

LM conceptualized the study; led the design, analysis, and interpretation of data; and drafted the manuscript. SH contributed substantively to analysis and interpretation of data and writing of the manuscript. DJD contributed substantively to analysis and interpretation of data and writing of the manuscript, VT contributed substantively to conceptualization of the study and interpretation of data, and provided critical editorial input to the manuscript. NRC contributed substantively to conceptualization of the study and interpretation of data, and provided critical editorial input to the manuscript. All authors read and approved the final manuscript.
